# Estimation of Tau and Phosphorylated Tau_181_ in Serum of Alzheimer’s Disease and Mild Cognitive Impairment Patients

**DOI:** 10.1371/journal.pone.0159099

**Published:** 2016-07-26

**Authors:** Shashank Shekhar, Rahul Kumar, Nitish Rai, Vijay Kumar, Kusum Singh, Ashish Datt Upadhyay, Manjari Tripathi, Sadanand Dwivedi, Aparajit B. Dey, Sharmistha Dey

**Affiliations:** 1 Department of Biophysics of All India Institute of Medical Sciences, New Delhi, 110029, India; 2 Department of Geriatric Medicine of All India Institute of Medical Sciences, New Delhi, 110029, India; 3 Department of Neurology of All India Institute of Medical Sciences, New Delhi, 110029, India; 4 Department of Biostatistics of All India Institute of Medical Sciences, New Delhi, 110029, India; Biomedical Research Foundation, UNITED STATES

## Abstract

The elevated level of cerebrospinal fluid (CSF) Tau and phosphorylated Tau_181_ (p-Tau_181_) proteins are well established hallmarks of Alzheimer’s disease (AD). Elevated level of p-Tau_181_ can differentiate AD from other neurodegenerative disease. However, the expression level of these proteins in serum of AD patient is not well set up. This study sought to evaluate the level of Tau and p-Tau_181_ in serum of AD, and mild cognitive impairment (MCI) patients for an alternative approach to establish protein-based markers by convenient way. Blood samples were collected from 39 AD patients, 37 MCI patients and 37 elderly individuals as controls. The levels of Tau and p-Tau_181_ in the serum of the different groups were measured by label free real time Surface Plasmon Resonance technology by using specific antibodies, and were further confirmed by the conventional western blot method. An appropriate statistical analysis, including Receiver Operating Characteristic (ROC), was performed. The concentrations of serum Tau and p-Tau_181_ were significantly higher (p<0.00001) in AD (Tau; 47.49±9.00ng/μL, p-Tau_181_; 0.161±0.04 ng/μL) compared to MCI (Tau; 39.26±7.78 ng/μL, p-Tau_181_; 0.135±0.02 ng/μL) and were further higher compared to elderly controls (Tau; 34.92±6.58 ng/μL, p-Tau_181_; 0.122±0.01 ng/ μL). A significant (p<0.0001) downhill correlation was found between Tau as well as p-Tau_181_ levels with HMSE and MoCA score. This study for the first time reports the concentration of Tau and p-Tau_181_ in serum of AD and MCI patients. The cutoff values of Tau and p-Tau_181_ of AD and MCI patients with sensitivity and specificity reveal that serum level of these proteins can be used as a predictive marker for AD and MCI.

## Introduction

Alzheimer’s disease (AD) is neurodegenerative disorder which causes a progressive dementia that currently affects over 35.6 million individuals worldwide and it is expected that it will be more than triple by 2050[1]. In 2010, 3.7 million Indians were demented and the total societal costs was about 14,700 crore. While the numbers are likely to be double by 2030, and cost would increase three times [2]. This imitates the socioeconomic burden of AD. There is no cure or disease-modifying therapies, and this may be due to our incapability to diagnose the disease before it has progressed to produce evident memory loss and functional decline.The biomarkers of disease will be critical for the development of disease-modifying or even preventive therapies. Unfortunately, the current available biomarkers for AD are either CSF based or imaging based which is either invasive, time consuming or highly expensive [3].

The well-known pathological hallmarks of Alzheimer’s disease (AD) are extracellular neurotoxic amyloidbeta peptide (Aβ peptide) laden plaques and intracellular neurofibrillary tangles (NFT) of hyperphosphorylated Tau [4]. Tau protein is abundant in neurons mainly active in the distal portion of axon of the central nervous system which stabilize microtubules. Tau protein controls the stability of microtubule by two ways; isoforms and phosphorylation [5]. The excessive phosphorylations of Tau detach the protein from microtubule and thus destabilize it [6]. The over expression of Tau and phosphorylated Tau in cerebrospinal fluid (CSF) of AD patients is well established [7]. Some recent studies evaluated plasma Tau levels in AD. They have reported a significant over expressed plasma Tau in AD and mild cognitive impairment (MCI) patients compared to healthy elderly [8, 9]. However, p-Tau_181_, which is more specific for AD, [10] has not been determined in blood of AD patients. Identifying protein marker in blood has many advantages over CSF as it is easily available from elderly AD patients in clinic or in home visits. Lumber puncture of elderly patients are quite often difficult task. Blood based markers are reliable for diagnostic and prognostic marker for early detection and follow up patients. Approximately 500 mL of CSF are absorbed into the blood every day; plasma may offer the rich source for biomarker in diseased condition. Moreover, damage to the blood brain barrier is a well-established event in this disease [11]. Keeping the above mentioned points and the socioeconomic burden of AD in mind, this study estimated the concentration of Tau and phosphorylated Tau (p-Tau_181_) in the serum of AD, MCI and normal elderly control by Surface Plasmon Resonance Technology (SPR), and was further validated by western blot experiment to explore the protein as marker for AD.

## Materials and Method

### Ethics

The Ethics Committee of All India Institute of Medical Sciences (AIIMS) approved the study protocol ((IESC/T-439/23.12.2014). Informed consent in writing was obtained from controls, patients or their attendants (if incapable of making a signature).

### Patients, controls and method

In this case control study of 113 subjects; 39 patients with AD, 37 patients with MCI and 37 elderly controls; were recruited from the Geriatric Medicine Memory Clinic and Neurology OPD of All India Institute of Medical Sciences, New Delhi, India. The AD and MCI patients with other comorbidities were excluded. The study was carried out as per the guidelines of Ethics Committee. The diagnosis of dementia involved a two-step diagnostic process. Older subjects with subjective cognitive complaints were first screened for cognitive impairment with Hindi Mental State Examination scale (HMSE; scores <24 out of 30) [12] and Montreal Cognitive Assessment (MOCA; score <22 out of 30) [13]. Subsequently, they were subjected to detailed evaluation including neurological examination, brain imaging, routine laboratory tests to exclude reversible causes of dementia, assessment of activities of daily living; and neuro-psychological testing (Clinical Dementia Rating Scale and the Blessed Dementia rating Scale) for categorization of dementia. AD was diagnosed as per NINCDS-ADRDA criteria [14]. MCI was diagnosed in presence of MoCA scores 18 to 26, respectively; and independence in activities of daily living. Older individuals above 60 years of age in good health (no obvious disease or disability in clinical examination) with normal HMSE and MoCA scores were invited to participate in the study as controls.

### Serum separation

Two milliliter (mL) of blood was collected from each participant by vein puncture in sterile vacutainer under strict aseptic conditions. The samples were kept at room temperature for 30–40 mins to clot. The clotted blood was centrifuged for 20 mins at 3000rpm to separate serum. Immediately after serum separation serum aliquots were preserved at -80°C for future use.

### Evaluation of Tau and phosphorylated Tau

#### By SPR

The concentrations of Tau and p-Tau181 protein were measured by SPR. All SPR measurements were performed at 25°C using the BIAcore-3000 [Wipro, GE Healthcare, Uppsala, Sweden] which is a biosensor-based system for real time specific interaction analysis. The goat IgG against Tau and rabbit IgG against p-Tau_181_ [Santa Cruz Biotechnology, CA] of human origin was immobilized on two different flow cells of CM5 sensor chip using the amine coupling kit [GE Healthcare, Sweden]. The system was equilibrated with running buffer; HBS-EP buffer (10 mM HEPES pH 7.4, 150 mM sodium chloride, 3 mM EDTA, 0.005% polysorbate 20), and maintained at a flow rate of 5 ml/min. The experimental flow cell dextran was activated using 1:1 volume mixture (110 μl each) of N-ethyl-N’-3 diethylaminopropylcarbodiimide (EDC) (75 μg/μl) and N-hydroxysuccinimide (NHS) (11.5 μg/μl). Antibody was diluted to 100 μg/ml in 10 mM sodium acetate (pH 5.0) and injected over the activated chip surface. Unreacted groups were blocked by ethanolamine (pH 8.5).

Different concentrations of purified recombinant human Tau protein and phosphopeptide corresponding to phosphorylated threonine 181 of isoform1 of human Tau were passed over the immobilized Tau and p-Tau_181_ antibodies on respective flow cells and corresponding resonance units (RU) were obtained. Tau protein was cloned, expressed and purified in bacterial system. Phosphopeptide was synthesized by using solid phase peptide synthesis as previously described [6]. The standard curve was prepared by plotting different concentration of Tau and p-Tau_181_versus corresponding RU obtained.

Similarly, serum samples were diluted (1:70) with HBS-EP buffer and passed over the immobilized antibodies in respective flow cells. The RU for each sample was recorded and the concentration of Tau and p-Tau_181_ in the serum of patients and elderly control was derived from the standard curve. All the experiments were done in triplicates.

#### By Western blot experiment

To confirm the presence of Tau and p-Tau in serum using western blotting, samples were prepared by depleting the albumin according to the manufacturer’s protocol [G Biosciences, St. Louis, USA]. Three serum samples from each group were selected randomly for western blot experiment. Total protein concentration was determined using Bicinchoninic acid assay (BCA) with bovine serum albumin as standard. Total protein (30 μg) was separated by sodium dodecyl sulfate polyacrylamide gel electrophoresis (SDS–PAGE). After the completion of SDS-PAGE, protein was transferred to polyvinylidene difluoride (PVDF) membranes (MDI Membrane Technologies, India). The unoccupied space on the PVDF membrane was then blocked with 5% nonfatty dry milk prepared in TBS (10 mM Tris pH 7.5, 150 mM NaCl) for 2h at room temperature and consequently incubated with primary antibodies diluted with TBS at 4°C overnight. Following antibodies and titers were used: primary goat antihuman Tau IgG (1:400), primary rabbit antihuman p-Tau_181_IgG (1:400). After proper washing with TBS-T (20 mM Tris pH7.5, 500 mM NaCl, 0.05% Tween 20), membranes were incubated with Horse Radish Peroxidase (HRP) conjugated secondary antibodies; goat anti rabbit IgG (1:4000) [GenScript, Piscataway, NJ], and donkey anti goat IgG (1:4000)[Santa Cruz Biotechnology, CA], at room temperature for 1h. After washing with TBS-T, bands were visualized by Enhanced Chemiluminescent System [Thermo Scientific, Rockford, IL]. The density of the bands obtained was determined using the Quantity-one1-D-analysis software [Bio-Rad Laboratories, Hialeah FL].

### Statistical Analysis

The data were analyzed by IBM SPSS statistics for Windows, Version 20.0 and Graphpad Instat3 software, presented in mean (SD) and frequency percentage. Continuous variables were compared among using one-way ANOVA followed by post hoc comparison using the Bonferroni test and chi-square was used for categorical variable. Correlation among the two continuous variables was calculated using Cary Pearson and Spearman correlation coefficient. Correlation coefficient was calculated for serum levels of Tau and p-Tau_181_ with HMSE, MoCA scores and between Tau and p-Tau_181_. Analysis of covariance was carried out to see the difference among the groups after adjusting gender, age, living status and education. Receiver Operating Characteristic (ROC) analysis was carried out to find the cutoff concentration of Tau and p-Tau_181_. P-value (p<0.05) was taken as statistically significant. The power of the study group was 98%.

## Results

### Demographic Data

A significant difference was observed in the median HMSE score, median MoCA score, education and gender between the groups (Table 1). Among 3 study groups the percentage of male was significantly higher. It was 51.21% in case of AD, 91.89% MCI and 70.21% elderly controls while, the percentage of female was 48.7, 8.10 and 29.72, respectively (p = 0.001). Maximum subjects in the study group of AD, MCI and elderly control were urban, i.e. 64.10%, 72.97% 67.56%, and the rural were 35.8 9, 27.02 and 34.43, respectively (p = 0.704). The education level is categorized in to 4 groups according to year of education in all the study groups i.e. 0–5, 6–10, 11–15 and 16+ in years. In case of 0–5 years of educated group: AD 28.20%, MCI 13.51% and elderly control 18.91%, in case of 6–10 years: 35.89%, 13.51% and 21.62%, in case of 11–15 years: 25.64%, 21.62% and 43.24%, and in case of 16+: 10.25%, 51.35% and 16.21%, respectively (p = 0.001). Analysis of covariance was applied and adjusted mean, standard error (SE) and p-value was reported after adjusting gender and education between three groups (Table 2).

**Table 1:** Demographic data of patients and controls.

	AD (n = 39)	MCI (n = 37)	Control (n = 37)	P-value
Sex				0.001
Male	20	34	26	
Female	19	3	11	
Average Age (±SD)	65.58±7.1	68.37±7.8	68.69±5.9	0.149
Average Disease duration in years (±SD)	3.11±1.37	1.35±0.8	---	>0.0001
Living status				0.706
Rural	14	10	12	
Urban	25	27	25	
Education in years				0.001
0–5	11	5	7	
6–10	14	5	8	
11–15	10	8	16	
16+	4	19	6	
HMSE, median with min-max (out of 30)	12(0–25)	24(17–28)	29(27–30)	>0.0001
MoCA median with min-max (out of 30)	8(0–21)	21(16–26)	28(26–30)	>0.0001

**Table 2:** Level of serum Tau and p-Tau_181_in study groups by SPR.

	AD (n = 39)	MCI (n = 37)	Control (n = 37)	P-value	Post-hoc comparison	
**Tau (ng/μL)**						P-value
Unadjusted mean ±SD	47.49±9	39.26±7.78	34.92±6.58	<0.0001	AD vs control	<0.001
					AD vs MCI	<0.001
					MCI vs control	0.059
*Adjusted mean ±SE	47.39±1.31	39.27±1.38	35.02±1.31	<0.001	AD vs control	<0.001
					AD vs MCI	<0.001
					MCI vs control	0.08
**p-Tau181 (ng/μL)** Unadjusted mean ±SD						
	0.161±0.04	0.135±0.02	0.122±0.01	<0.0001	AD vs control	<0.0001
					AD vs MCI	<0.002
					MCI vs control	0.233
*Adjusted mean ±SE	0.157±0.005	0.139±0.005	0.124±0.005	<0.001	AD vs control	<0.0001
					AD vs MCI	0.065
					MCI vs control	0.131

*Covariates appearing in the model are evaluated at the following: sex, age, education, and living status.

### Estimated Tau and phosphorylated Tau in serum

#### By SPR analysis

**Fig 1:**
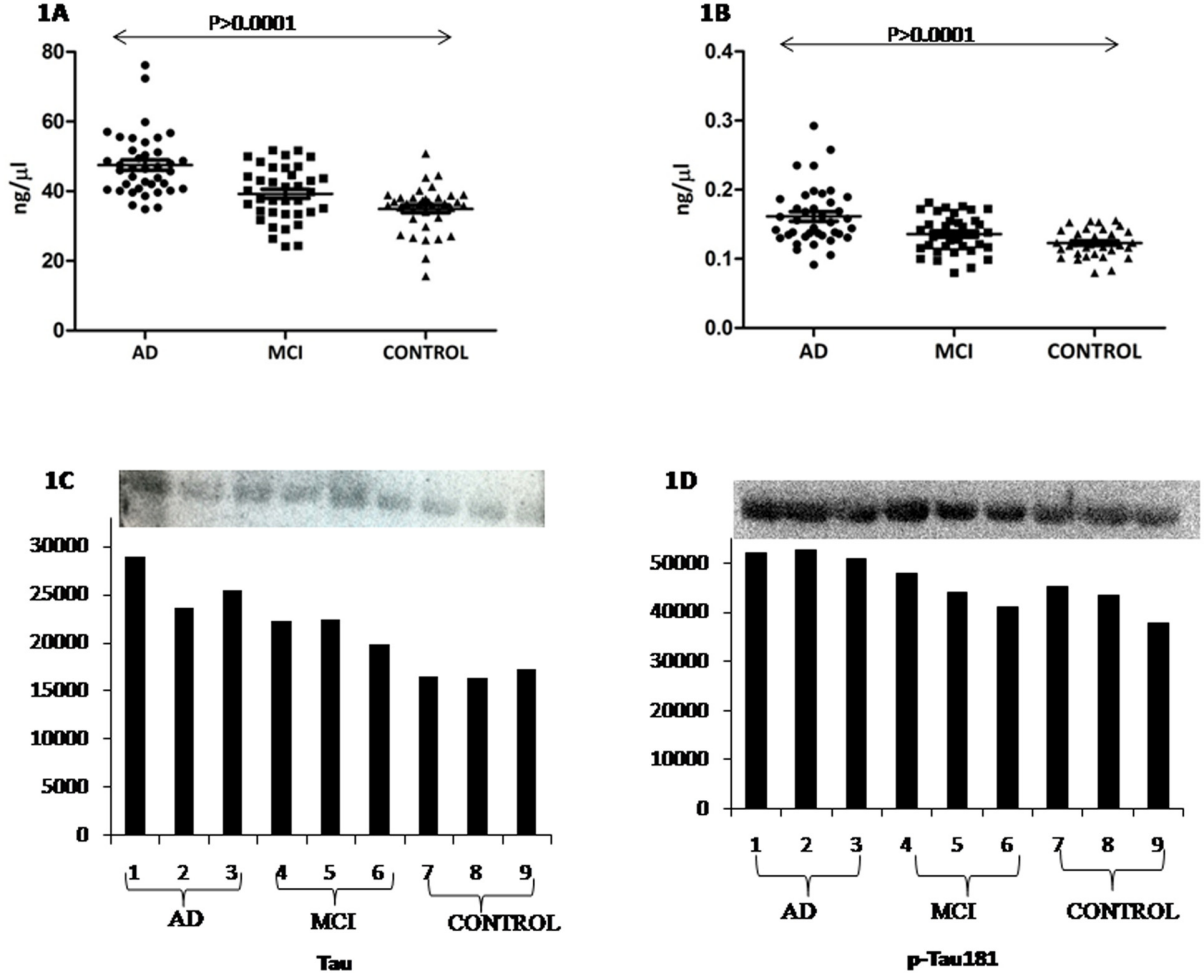
Scatter graph showing the serum level of Tau (A) and p-Tau181 (B) in AD, MCI and elderly control.

**Fig 2:**
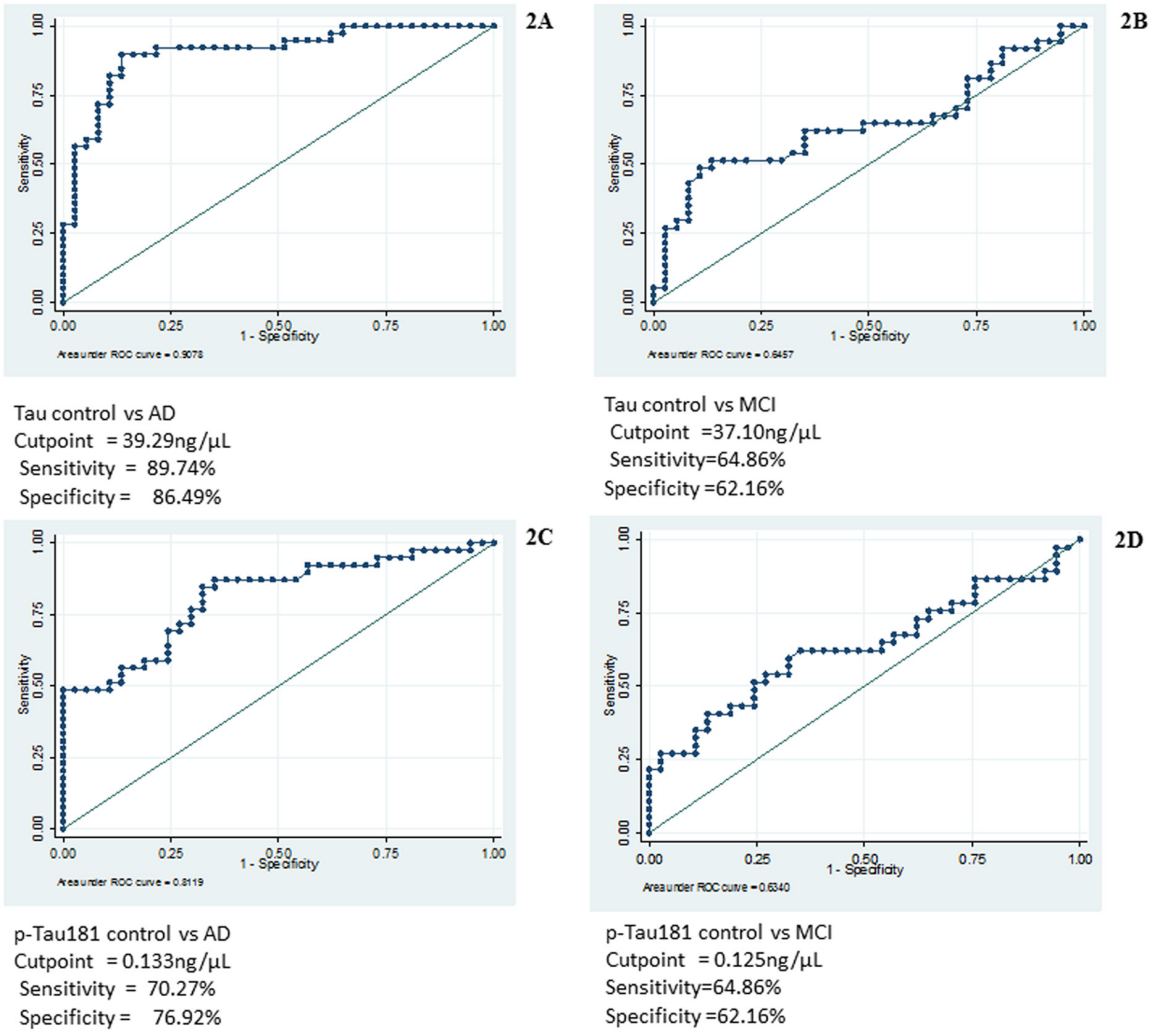
ROC analysis showing area under curve, threshold value with sensitivity and specificity, for Tau: (A) AD vs control (B) MCI vs control for p-Tau_181_: (C) AD vs control (D) MCI vs control.

The response unit (RU) of immobilized antibodies; Tau and p-Tau_181_ were 5944.8 and 5903.6, respectively. A standard curve was plotted with RU obtained from the sensorgram with different concentrations of pure recombinant Tau and p-Tau_181_ phosphopeptide. The binding of the ligands (Tau protein and p-Tau_181_ phosphopeptide) were in the linear range. The concentration (mean with SD) of Tau (47.49±9.00 ng/μL, 95% CI 44.58–50.71) in AD patients was significantly (p<0.00001) higher than that of MCI patients (39.26±7.78 ng/μL, 95% CI 36.67–41.86) and elderly control (34.92±6.58 ng/μL, 95% CI 32.73–37.13). The concentration (mean with SD) of p-Tau_181_ was also found to be significantly higher, in case of AD (0.161±0.04 ng/μL, 95% CI 0.147–0.175) as compared to MCI (0.135±0.02 ng/μL, 95% CI 0.126–0.144) and elderly control (0.122±0.01 ng/μL, 95% CI 0.116–0.129) (p<0.00001) (Fig 1A & 1B). After adjustment for following covariates: gender, age, and education the concentrations (mean with SE) of Tau and p-Tau_181_ were significantly higher in AD patients (Tau 47.39±1.34 ng/μL, p-Tau_181_ 0.157±0.005 ng/μL) as compared to that of MCI patients (Tau 39.27±1.38 ng/μL, p-Tau_181_ 0.139±0.005 ng/μL) and elderly controls (Tau 35.02±1.31 ng/μL, p-Tau_181_ 0.124±0.005 ng/μL). ROC analysis revealed that area under curve (AUC) for Tau was 0.907 in case of AD vs control. The cutoff value of Tau was 39.29 ng/μL with sensitivity of 86.49% and specificity of 89.74% to detect AD patients (Fig 2A). For MCI vs control the AUC for Tau was 0.645, the cutoff value was 37.10 ng/μL with sensitivity of 64.86% and specificity of 62.16% to detect MCI patients (Fig 2B).

In case of p-Tau_181_, AUC was 0.811 for AD vs control, the cutoff concentration was 0.133 ng/μL with sensitivity of 70.27% and specificity of 76.92% (Fig 2C). In case of MCI vs control, the AUC was 0.634, the cutoff value was 0.125 ng/μL with sensitivity 64.86% and specificity 62.16% (Fig 2D).

A significant moderate and downhill linear correlation was found between Tau levels and HMSE score (-0.4273) as well as MoCA score (-0.4613). This same observation was obtained in the case of p-Tau_181_ levels (-0.3899) with HMSE score (-0.3962) as well as MoCA score (p<0.00001). There was a significant strong uphill correlation (0.6857) between Tau and p-Tau_181_ levels (p<0.00001). After the analysis of covariance there was significant difference in the serum levels of Tau and p-Tau_181_ among study groups.

#### By Western Blot analysis

The western blot experiment showed the presence of Tau and p-Tau_181_ in the serum of all study groups with higher density for both p-Tau_181_and Tau bands in case of AD and MCI patients as compared to controls (Fig 1C and 1D).

## Discussion

The concept of blood based biomarkers for AD is alluring, and these could be put to many uses, such as screening, diagnosis, risk assessment, and as an aid to drug development in clinical trials. Various studies have previously been conducted for candidate biomarkers in blood, such as Aβ protein, Aβ autoantibodies, platelets, amyloid precursor protein (APP) isoforms, inflammation (cytokines), oxidative stress (vitamin E, isoprostanes) vascular diseases (homocysteine, lipoprotein A) and lipid metabolism (Apo lipoprotein E, 24S-Hydroxy-cholestrol) [15]. Due to the lack of sensitivity and specificity most of these were fail to be potent blood based biomarker. As per our knowledge, we are here first time using the SPR technique to quantify serum Tau and p-Tau_181_ in AD and MCI patient groups. Till now these two proteins are found to be CSF based markers in clinical trials [16]. In the present study, the concentration of both serum Tau and p-Tau_181_ were significantly higher in the case of AD compared to MCI and control healthy elderly.

Though CSF Tau and p-Tau_181_ of AD patients determined by ELISA method are well established, but still the sensitivity and specificity vary in different studies [7, 8, 9]. Recently Ming-Jang Chiu et al., 2014 reported the elevated level of plasma Tau and negative association with logical memory, visual reproduction, and volume of total gray matter, hippocampus and gray matter density [9]. High Tau level has been associated with progression of AD with a high mortality rate. Tau protein interacts with tubulin and maintains the stability of microtubule by their phosphorylation state. There are 79 Ser and Thr phosphorylation sites in Tau [5], the phosphorylation state of these sites are regulated by Tau kinases and phosphatases [17]. The failure of this regulation causes hyperphosphorylation of Tau on these specific sites, which finally results in the formation of NFTs and neuronal cell death [18]. It has been observed that in the case of AD patients, the formation of NFTs in the brain was correlated with increased levels of hyperphosphorylation at p-Tau_181_ or p-Tau _231_ [19]. High p-Tau_181_ has also been associated with faster progression of MCI to AD [10] and rapid cognitive decline in AD [20]. It has also been reported that increased phosphorylation at p-Tau_181_ has correlation with hippocampal atrophy [21]. CSF p-Tau_181_ concentration was determined in previous studies [22]. Here, we measured elevated level of p-Tau_181_ in serum of AD patients, MCI patients compared to elderly controls by SPR technology, which is a label free real time method. In the previous study we have reported down regulated serum sirtuin protein in AD compare to elderly control by SPR technology [23]. The cutoff concentrations determined by ROC analysis were highly sensitive and specific to differentiate AD and MCI from control. The high sensitivity and specificity of these proteins may be useful to diagnose the disease with high accuracy by avoiding false positive and false negative results.

A significant correlation (p<0.00001) existed between serum Tau and p-Tau_181_ concentration with HMSE/MoCA scores. The moderate negative correlation of protein with the HMSE and MoCA score indicates that the serum levels of Tau and p-Tau_181_ increases with decreasing HMSE and MoCA score. This finding shows the relationship between the serum protein levels and the neuropsychological parameters which can be valuable in the clinical setup for the diagnosis of AD and MCI. These results can be used to detect patients in early dementia. The strong and positive correlation between Tau and p-Tau_181_ in serum of AD, MCI and elderly controls indicates that as the level of Tau increases, the level of p-Tau_181_ will increase simultaneously.

In conclusion, the serum Tau and p-Tau_181_ were capable of differentiating between elderly controls from AD and MCI patients and also AD from MCI patients. Hence, serum p-Tau_181_ can serve as a predictive protein marker for MCI and AD patients and can also work as prognostic marker during follow up of patients in the future. The correlation between CSF and serum p-Tau_181_ is remain to be future prospective.
